# Real-Time Orbit Determination of Micro–Nano Satellite Using Robust Adaptive Filtering

**DOI:** 10.3390/s24247988

**Published:** 2024-12-14

**Authors:** Jing Chen, Xiaojun Jin, Cong Hou, Likai Zhu, Zhaobin Xu, Zhonghe Jin

**Affiliations:** 1 Huanjiang Laboratory, School of Aeronautics and Astronautics, Zhejiang University, Hangzhou 310027, China; 22224023@zju.edu.cn (J.C.); 12024045@zju.edu.cn (C.H.); 12324045@zju.edu.cn (L.Z.); zjuxzb@zju.edu.cn (Z.X.); jinzh@zju.edu.cn (Z.J.); 2 Zhejiang Key Laboratory of Micro-Nano Satellites Research, Hangzhou 310027, China

**Keywords:** micro–nano satellites, GPS, orbit determination accuracy, robust estimation, satellite navigation

## Abstract

Low-performing GPS receivers, often used in challenging scenarios such as attitude maneuver and attitude rotation, are frequently encountered for micro–nano satellites. To address these challenges, this paper proposes a modified robust adaptive hierarchical filtering algorithm (named IARKF). This algorithm leverages robust adaptive filtering to dynamically adjust the distribution of innovation vectors and employs a fading memory weighted method to estimate measurement noise in real time, thereby enhancing the filter’s adaptability to dynamic environments. A segmented adaptive filtering strategy is introduced, allowing for flexible parameter adjustment in different dynamic scenarios. A micro–nano satellite equipped with a miniaturized dual-frequency GPS receiver is employed to demonstrate precise orbit determination capabilities. On-orbit GPS data from the satellite, collected in two specific scenarios—slow rotation and Earth-pointing stabilization—are analyzed to evaluate the proposed algorithm’s ability to cope with weak GPS signals and satellite attitude instability as well as to assess the achievable orbit determination accuracy. The results show that, compared to traditional Extended Kalman Filters (EKF) and other improved filtering algorithms, the IARKF performs better in reducing post-fit residuals and improving orbit prediction accuracy, demonstrating its superior robustness. The three-axes orbit determination internal consistency precision can reach the millimeter level. This work explores a feasible approach for achieving high-performance orbit determination in micro–nano satellites.

## 1. Introduction

In conventional satellite navigation applications, P-code (Precise Code) is extensively employed as a high-precision navigation signal, particularly for military and demanding scientific endeavors, due to its remarkable accuracy; however, as a military-grade code, P-code necessitates intricate decryption and possesses a high pseudocode rate, rendering it challenging to implement on resource- and power-constrained micro- and nano-satellite platforms. Conversely, the C/A (Coarse/Acquisition Code) is characterized by its simplicity, low power consumption, and broad compatibility without encryption, making it well-suited for systems with limited resources. Nonetheless, its inferior pseudocode accuracy, combined with the diminutive antenna sizes of micro- and nano-satellites, results in weakened reception of navigation signals, adversely affecting data quality and stability. This creates substantial challenges for achieving precise orbit determination. Thus, enhancing the robustness of orbit determination algorithms to bolster system resilience and ensure accuracy has become a critical concern [1,2,3].

Moreover, satellites frequently need to execute maneuvers such as pitching, slow rotation, scanning, and stabilization while performing specific tasks. During these maneuvers, the satellite may deviate from zenith and exhibit angular velocity, which detrimentally impacts navigation signal reception and presents additional challenges for orbit determination. Consequently, robust algorithms are essential for ensuring system resilience in these scenarios [4,5,6,7,8,9,10,11].

Given these complexities, traditional Extended Kalman Filtering (EKF) algorithms often prove inadequate for the orbit determination demands of micro- and nano-satellites. EKF’s reliance on linearization assumptions and its computational intricacies limit its efficacy in addressing highly nonlinear and dynamic changes. In contrast, robust adaptive algorithms can estimate noise variance in real time and adeptly manage outliers, facilitating adaptive adjustments to estimation methodologies and parameters in complex operational contexts. This adaptability enables precise and reliable orbit determination, even amidst constrained GPS receiver capabilities and stringent mission requirements [12,13,14,15].

An enhanced robust adaptive hierarchical filtering strategy and algorithm are proposed, specifically designed to address the distinct characteristics of GPS receivers on micro–nano satellites as well as the operational challenges encountered in their environments. The orbit determination performance is validated using data obtained from a micro–nano satellite equipped with a miniaturized GPS receiver, operating in orbit under two distinct conditions, slow rotation and Earth stabilization, thereby ensuring the authenticity and relevance of the results. The evaluation demonstrates the robustness and precision of the proposed strategy and algorithm in ensuring accurate orbit determination, particularly in scenarios involving weak GPS signals and satellite attitude instability.

## 2. Materials and Methods

The standard Kalman filtering algorithm assumes that both the observation measurements and the state prediction vector follow a normal distribution. However, in actual on-orbit missions, the observation vector and the state model may exhibit anomalies, leading to significantly large errors in the filtering process. Therefore, enhancing filtering algorithms to improve their robustness has become a crucial area of research. By integrating techniques such as robust statistics, adaptive filtering, and outlier detection, these improved algorithms can effectively mitigate the impact of non-Gaussian noise and unexpected disturbances, ensuring more accurate and reliable state estimation in challenging environments.

### 2.1. Filters Methods

Improved Kalman filtering typically refers to the extension and enhancement of the basic Kalman filtering algorithm. This is achieved by incorporating adaptive filtering techniques and robust statistical methods to enhance estimation accuracy, particularly in addressing complexities such as nonlinear dynamics and non-Gaussian noise. The objective is to create a more resilient filtering framework capable of effectively managing these challenging conditions.

#### 2.1.1. Robust Kalman Filtering

Based on the Extended Kalman Filter (EKF), the Robust Kalman Filter (RKF) algorithm is constructed under the assumption that the observation vector $L_{k}$ and the state prediction vector ${\bar{X}}_{k}$ at epoch $t_{k}$ both follow a contaminated normal distribution. The RKF employs M-estimation for both $L_{k}$ and ${\bar{X}}_{k}$, redistributing the weights of the observations. The reconstructed state estimate and its covariance matrix are recursively updated as follows:

(1) $$\begin{array}{c} {\hat{x}}_{k}={\left(H_{k}^{T}{\bar{P}}_{k}H_{k}+{\bar{P}}_{{\hat{x}}_{k|k-1}}\right)}^{-1}(H_{k}^{T}{\bar{P}}_{k}L_{k}\\ +{\bar{P}}_{{\hat{x}}_{k|k-1}}{\hat{x}}_{k|k-1}) \end{array}$$

(2) $$\sum {\hat{x}}_{k}={\left(H_{k}^{T}\bar{P}H_{k}+{\bar{P}}_{{\hat{x}}_{\left(k|k-1\right)}}\right)}^{-1}$$

where

${\hat{x}}_{k}$ is the state estimate at the current time k;

$H_{k}$ is the observation matrix that relates the state to the observation vector;

${\bar{P}}_{k}$ is the observation noise covariance matrix;

$L_{k}$ is the observation vector.

#### 2.1.2. Innovative Adaptive Kalman Filtering

In the context of standard observations, the innovation sequence is expected to follow a Gaussian white noise distribution, with its elements being mutually independent. However, inaccuracies in the process noise and measurement noise can lead to filtering divergence. To address this issue, the Innovative Adaptive Kalman Filter (IAKF) simultaneously adjusts the state-process-noise matrix and the measurement-noise matrix based on the observed values, achieving adaptive noise covariance parameter estimation.

(3) $${\hat{R}}_{k}={\hat{C}}_{{\bar{V}}_{k}}+H_{k}P_{k|k-1}H_{k}^{T}$$

(4) $${\hat{Q}}_{k}\approx K_{k}{\hat{C}}_{k}K_{k}$$

where ${\hat{C}}_{\bar{V_{k}}}$ represents the predicted value of the innovation vector, which can be calculated using a sliding window approach to average recent observations. However, due to the reliance on the innovation vector that incorporates errors from the state prediction vector (known as Innovation Adaptive Estimation, IAE), the method faces challenges in effectively addressing errors in the dynamical model; moreover, it necessitates that the residual sequence is of the same dimension and distribution.

For the orbit determination of low Earth orbit (LEO) satellites, the frequent changes in satellite tracking conditions in real-world scenarios hinder the ability to maintain stable usage across the entire observation arc. This variability complicates the effective application of the IAKF as it becomes difficult to ensure consistent performance in dynamically changing environments. As a result, there is a pressing need for adaptive filtering strategies that can accommodate these fluctuations and enhance robustness in the estimation process.

#### 2.1.3. Sage–Husa Adaptive Filtering

The Sage–Husa Kalman Filter (SKF) addresses the limitations of traditional adaptive filtering methods by providing a real-time estimate of the measurement noise variance matrix based on the posterior residual error sequence (RAE). This approach circumvents the potential drawback of matrix indefiniteness that can occur in filtering algorithms utilizing Innovation Adaptive Estimation (IAE) while also improving the handling of anomalous observations.

The residual sequence and the corresponding measurement noise variance matrix are expressed as follows:

(5) $$V_{k}=(L_{k}-H_{k}{\hat{x}}_{k})$$

(6) $${\hat{R}}_{k}={\hat{C}}_{V_{k}}+H_{k}P_{k}H_{k}^{T}$$

### 2.2. Improved Adaptive Robust Filtering with Hierarchical Strategy

The Sage–Husa adaptive filtering algorithm (SKF) primarily enhances filter performance through the estimation of time-varying noise variances; however, while SKF optimizes the handling of complex noise, it does not sufficiently address the non-Gaussian characteristics or the presence of outliers in the noise. Consequently, SKF still lacks effective resistance when faced with extreme anomalies in the measurement noise. To overcome this limitation, we propose an improved method based on SKF that incorporates an adaptive factor and employs a three-segment robust (IGG3) weighting function to manage anomalous observations. Specifically, we utilize the IGG3-based weighting function to weight the innovation vector $\bar{V_{k}}$, thereby identifying and suppressing outliers in both the dynamic model errors and measurement errors. This enhancement is aimed at improving filtering performance within the framework of SKF [16].

The Improved Adaptive Robust Kalman Filter (IARKF) is derived from the SKF filter and maintains consistency in the processing of measurement values, which includes requirements for the same statistical distribution category, dimensionality, and probability distribution characteristics. Given the practical scenario of frequent changes in navigation satellites during on-orbit tasks, we introduce a hierarchical adaptive strategy. When changes in the number of tracked satellites are detected, the filter utilizes the Adaptive Robust Kalman Filter (ARKF) to address potential noise and error issues in a rapidly changing environment. Conversely, when the number of measurements of the same dimension reaches a minimum filtering window N, the filter upgrades to the Improved Adaptive Robust Kalman Filter (IARKF) to further enhance filtering performance.

In specific applications, particularly in the analysis of data from the in-orbit satellite, we found that the reliability of statistical filtering methods significantly decreases when the number of satellites is low. When the Geometric Dilution of Precision (GDOP) is excessively high, it indicates poor geometric distribution of the positioning satellites, which increases the uncertainty of the position estimates. Therefore, we implement a special strategy: when the GDOP exceeds a certain empirical threshold or the number of satellites involved in positioning falls below a certain value, the filter is downgraded to the Extended Kalman Filter (EKF) without adaptive inflation of the measurement noise matrix, prioritizing the robustness of the orbit determination. When the filter operates as ARKF or IARKF, the adaptive factor is selected based on the accuracy of the dynamic model to optimize filtering performance.

The equivalent posterior residual ${\hat{\bar{V}}}_{k(i)}$ can be expressed as follows:

(7) $${\hat{\bar{V}}}_{k(i)}=\left\{\begin{array}{ccc} {\bar{V}}_{k(i)} & , & \left|{\bar{V}}_{k(i)}\right|\leq k_{0}\\ \frac{{\bar{V}}_{k(i)}}{k_{1}} & , & k_{0}<\left|{\bar{V}}_{k(i)}\right|\leq k_{1}\\ 0 & , & \left|{\bar{V}}_{k(i)}\right|>k_{1} \end{array}\right.$$

where $k_{0}$ and $k_{1}$ are harmonic coefficients, typically with $k_{0}$ ranging from 0.8 to 1.5 and $k_{1}$ from 3 to 5. For this algorithm, we set $k_{0}$ = 1 and $k_{1}$ = 3.

In practical spaceborne high-dynamic environments, the sources of noise are complex and difficult to distinguish accurately. Traditional averaging methods exhibit significant lag when dealing with dynamic changes and outliers. Given that the process noise in dynamic model theoretical modeling is relatively precise, while outliers primarily occur during the measurement process, the Exponentially Weighted Moving Average (EWMA) method adjusts weights to replace the traditional multi-epoch averaging method for estimating the noise matrix. By assigning higher weights to more recent observations, this approach allows for recursive updates of the matrix, avoiding redundant computations. Compared to averaging data across epochs, it can respond more swiftly to sudden noise changes and outliers, thereby enhancing the identification of recent trends in the time series [17,18].

The weighting coefficient β satisfies the following:

(8) $$\sum_{i=1}^{k}b_{i}=1,b_{i-1}=b_{i}b,\;0<b<1$$

where b is the forgetting factor, typically taken to be between 0.95 and 0.995. In this study, we set b = 0.95. The weights decrease for data points farther from the current epoch and increase for those closer, with the weighting coefficients constructed as follows:

(9) $$\left\{\begin{array}{l} \beta_{i}=d_{k}b^{k-i}\\ d_{k}=\frac{1-b}{1-b^{k}},i-1,2,\cdot \cdot \cdot ,k \end{array}\right.$$

Thus, the equal-weighted measurement noise can be expressed as outlined below:

(10) $$\begin{array}{ll} {\hat{R}}_{k} & =\sum_{i=1}^{k}\beta_{i}(V_{i}V_{i}^{T}+H_{i}P_{i}H_{i}^{T})\\ & =\sum_{i=1}^{k}d_{k}b^{k-i}(V_{i}V_{i}^{T}+H_{i}P_{i}H_{i}^{T})\\ & =\sum_{i=1}^{k-1}d_{k}b^{k-i}(V_{i}V_{i}^{T}+H_{i}P_{i}H_{i}^{T})+\\ & \;\;\;\;d_{k}(V_{k}V_{k}^{T}+H_{k}P_{k}H_{k}^{T}) \end{array}$$

By updating the noise matrix through weighted averaging, the adaptive factor can be adjusted more rapidly, enhancing the IARKF’s capability in estimating measurement noise. This improvement boosts the overall accuracy and stability of the filter in estimating states under complex noise conditions in high-dynamic environments. The formulas involved in the IARKF improvements are summarized as follows:

(11) $$\begin{array}{l} \left\{\begin{array}{c} {\hat{x}}_{k|k-1}=\Phi_{k,k-1}{\hat{x}}_{k-1}\\ {\hat{P}}_{k|k-1}=\Phi_{k,k-1}{\hat{P}}_{k}\Phi_{k,k-1}^{T}+Q_{k-1} \end{array}\right. \end{array}$$

(12) $$\left\{\begin{array}{c} K_{k}=\frac{1}{\alpha_{k}}{\hat{P}}_{k|k-1}H_{k}^{T}{\left({\hat{R}}_{k}+\frac{1}{\alpha_{k}}H_{k}{\hat{P}}_{k|k-1}H_{k}^{T}\right)}^{-1}\\ {\hat{x}}_{k}={\hat{x}}_{k|k-1}+K_{k}\hat{\bar{V}}\\ {\hat{P}}_{k}=(I-K_{k}H_{k}){\hat{P}}_{k|k-1}{\left(I-K_{k}H_{k}\right)}^{T}+K_{k}{\hat{R}}_{k}K_{k}^{T} \end{array}\right.$$

In the Improved Robust Adaptive Kalman Filter (IARKF), two main optimizations have been implemented compared to the traditional Extended Kalman Filter (EKF).

First, the IARKF replaces the measurement noise and innovation vector in the traditional EKF with equivalent measurement noise ${\hat{R}}_{k}$ and equivalent innovation vector $\hat{\bar{V}}$. This optimization allows the filter to handle the non-Gaussianity of noise and outliers more effectively.

Second, the IARKF adopts the predicted state covariance matrix representation as the method for solving the adaptive factor. This approach can absorb minor perturbations in the dynamic model and does not require observation values to have the same dimensionality, thus maximizing coverage for full arc orbit determination. This optimization enables the filter to perform stable state estimation under a broader range of conditions, improving the system’s adaptability to dynamic changes.

When the number of satellites involved in positioning is sufficiently high, the size of the robust estimate ${\bar{x}}_{k}$ can be compared to the predicted value $x_{k,k-1}$ to determine the size of the adaptive factor, with the specific calculation formula as follows:

(13) $$\alpha =\left\{\begin{array}{c} \;1\;\;\;\;,\left|\Delta x_{k}\right|\leq c_{0}\\ \frac{c_{0}}{\left|\Delta x_{k}\right|}(\frac{c_{1}-\left|\Delta x_{k}\right|}{c_{1}-c_{0}}{)}^{2}\;,\;c_{0}<\left|\Delta x_{k}\right|<c_{1}\\ \;0\;\;\;,\;\left|\Delta x_{k}\right|>c_{1} \end{array}\right.$$

(14) $$\Delta x_{k}=\frac{\left\|{\bar{x}}_{k}-x_{k,k-1}\right\|}{\sqrt{tr\left\{P_{k,k-1}\right\}}}$$

where α is the adaptive factor; and $c_{0}$ along with $c_{1}$ are constants, typically with $c_{0}$ ranging from 0.8 to 1.5 and $c_{1}$ from 3 to 5. In this algorithm, we set $c_{0}$ = 1 while $c_{1}$ = 3. The notation tr denotes the trace of a matrix; and $\left\|\;.\;\right\|$ represents a norm operation.

**Figure 1:**
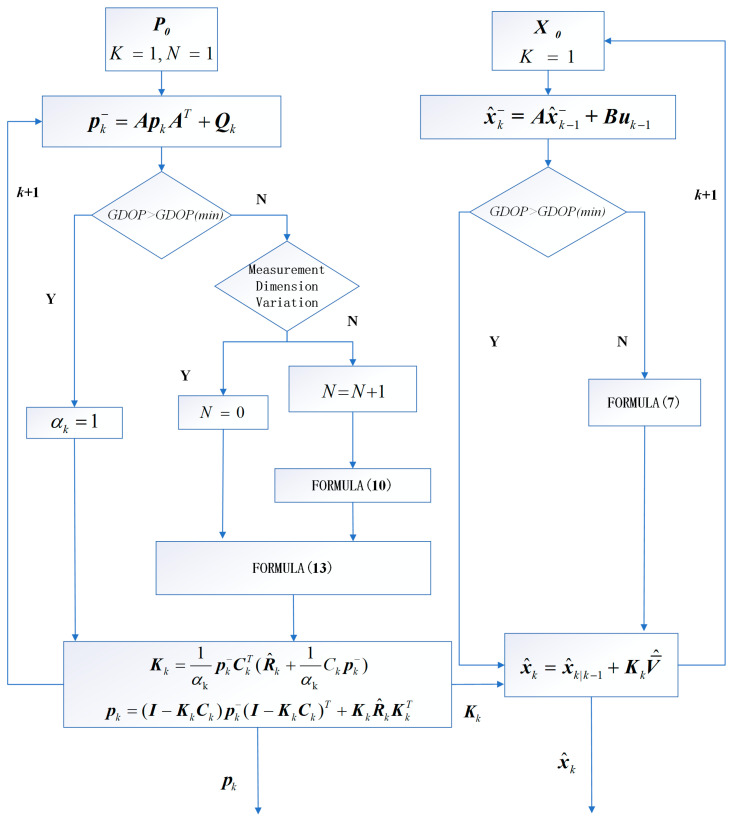
Flowchart of IARKF strategy.

In the process of determining the adaptive factor, a three-segment strategy is employed, dividing the adaptive factor into three main regions: the retention zone, the reduction zone, and the elimination zone. Specifically, the retention zone maintains the current weight of the adaptive factor, the reduction zone gradually decreases its weight, while the elimination zone reduces the weight to zero. These divisions are based on the error differences between the actual estimated values and the predicted values of the state variables. The adaptive factor is dynamically adjusted within the range of 0 to 1 based on this difference to optimize the system‘s response and enhance prediction accuracy. For additional details, please consult Figure 1. 

### 2.3. Orbit and Geodetic Settings

We employ a series of simplified dynamic models and data processing strategies to analyze satellite orbits. In gravitational field modeling, we use the JGM3 (20 × 20) model (Tapley et al., 1996), which accounts for the gravitational effects of the moon and the sun, while neglecting the influence of solid Earth, ocean, and polar tides. For orbit calculation, we adopt the N-body gravity method with slightly lower accuracy (Montenbruck and Pfleger, 2013), and the cylindrical shadow model for solar radiation pressure (Montenbruck et al., 2002). Additionally, the Harris–Priester model is used to describe atmospheric drag, for a detailed overview, readers may refer to Table 1.

In the reference system, we utilize the J2000/WGS84 coordinate system, and the IAU 1976/IAU-1980 simplified model is applied to handle precession and nutation. For GNSS data processing, we use the GPS L1C + L2C signals, process broadcast ephemerides using an undifferenced combination method, and apply floating-point ambiguity resolution. Ionospheric effects are eliminated using dual-frequency ionosphere-free techniques. All data are processed at a 1 Hz sampling rate.

The data used in this analysis were obtained from a satellite in continuous low orbit, which specifically operates in a sun-synchronous orbit at an altitude of approximately 500 km. It is equipped with a dual-mode, four-frequency GPS/BDS receiver developed independently, supporting L1C/A, L2C, B1I, and B3I frequency bands. The receiver features a miniaturized design, weighing only 100 g with a power consumption of 3.5 W. The satellite innovatively utilizes dual-frequency civilian GPS signals for orbit determination validation, aiming to explore technical pathways suitable for the precise orbit determination of micro–nano satellites.

This study focuses on the analysis of L1C/A and L2C frequency data from GPS. In the absence of external independent observation sources, intermediate data from the orbit determination process along with the satellite’s own orbit determination results are utilized for an internal consistency assessment. Initially, a systematic analysis of the residuals between the observed data and model predictions is conducted to evaluate the fitting accuracy of the observations. When the satellite dynamics model and the observation correction model align with actual conditions, the posterior residuals should approach the level of observation noise. GDOP is used as a supplementary metric to assess orbit determination accuracy, providing quantitative information on how the satellite geometric configuration affects positioning precision, thus aiding in the analysis of the reliability of satellite positioning accuracy. Additionally, we perform orbital overlap difference statistics to evaluate the stability and consistency of orbital predictions, offering further validation of the orbit determination accuracy [19,20].

Through a comprehensive analysis of the aforementioned data, we are able to thoroughly assess the intrinsic accuracy of the orbit determination results and provide theoretical support and data backing for further improvements to the orbit determination system. Table 2 provides additional information for readers’ reference.

## 3. Verification of the Slow Rotation Scenario for In-Orbit Satellite

A slow rotation scenario of the satellite is primarily used to validate the robustness of the algorithm presented in this paper. A set of receiver telemetry data from a micro–nano satellite operating in the slow rotation phase was selected (with an angular velocity of approximately 0.2°/s) for analysis. The total available arc length for the orbit determination in this dataset is 2.5 h, specifically consisting of two main data segments: the first segment spans from 18 July 2023 at 23:02:49 to the next day at 01:01:43, with an orbit determination arc length of approximately 2 h; the second segment runs from 18 July at 23:32:08 to the next day at 01:35:50, also with an orbit determination arc length of approximately 2 h. There is a 1.5-h overlapping arc segment between the two data segments.

### 3.1. GDOP

In the analysis of the time variation in GDOP (Geometric Dilution of Precision) for the GPS frequencies on the unique satellite, we examined the GDOP data for the L1C/A and L2C frequencies as well as their combined effects. It is clear that the GDOP value for the L2C frequency is higher than that for the L1C/A frequency. This phenomenon is primarily due to the close relationship between GDOP and the number and spatial distribution of the satellites. According to the official GPS website, as of 3 July 2023, the GPS system has only 25 satellites supporting the L2C frequency, most of which are Block IIR-M satellites launched after 2005. This insufficient coverage results in a higher GDOP value for the L2C frequency in actual observations. Detailed information regarding the status of the satellites broadcasting civilian signals can be found in Table A1 in Appendix A.

To enhance the orbit determination accuracy, this paper employs a dual-frequency ionospheric-free combination. This combination method relies on relatively few frequency points for actual observations when calculating GDOP. Specifically, for the GPS system, the GDOP of the dual-frequency ionospheric-free combination aligns closely with the GDOP at the L2C frequency. The reason is, when using the dual-frequency combination, the system’s observations are primarily constrained by satellites at the L2C frequency, which means that the GDOP value largely reflects the characteristics of the L2C GDOP. Consequently, the GDOP value of the dual-frequency ionospheric-free combination is consistent with that of the L2C frequency. This finding suggests that, although the L2C frequency theoretically provides the capability to correct for ionospheric delays, its limited coverage indicates that there is still room for performance improvement.

When the satellite is in a rotating state, its orientation is not aligned with the zenith, and it possesses a certain angular velocity, further affecting the number of observed satellites and GDOP. A comparison of the relationship between GDOP and the number of satellites reveals a strong negative correlation: when the number of positioning satellites is low, GDOP increases. In some epochs, the insufficient number of satellites leads to excessively high GDOP values, which increases the system’s sensitivity to errors. This means that even small measurement errors or noise can be amplified, resulting in larger residuals in the positioning results. This cumulative effect can significantly increase the posterior residuals when GDOP values fluctuate greatly, leading to more outliers.

It is worth noting that as the modernization of GPS progresses, the number of satellites supporting the L2C signal will gradually increase. This will effectively enhance the coverage of the L2C signal and improve the spatial distribution of the satellites, which means that the accuracy and reliability of positioning using the L2C frequency will be significantly improved.

**Figure 2:**
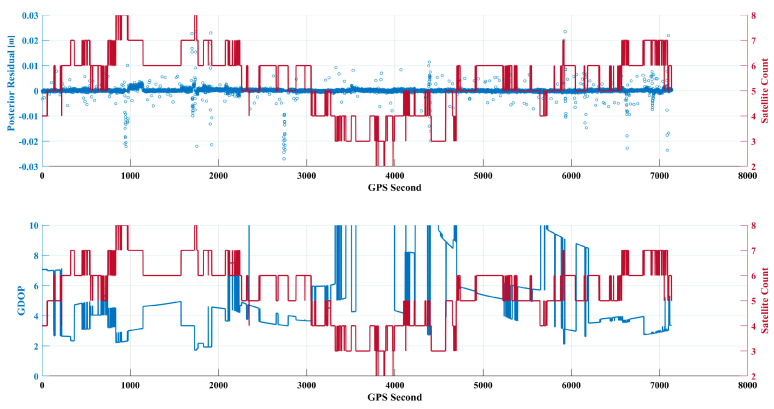
Comparison of posterior residuals, number of positioning satellites, and GDOP.

As shown in Figure 2, the proportion of epochs during which the micro–nano satellite has five or more navigation satellites in view is 75%. The average number of navigation satellites involved in positioning is 5.3, and the average GDOP value using the ionospheric-free combination is 2.5683, with a channel average posterior residual of 0.0083 m.

From the observations in the figures, it is evident that most of the posterior residuals are concentrated below 10 cm, although there are also several outlier residuals. Some of these outlier intervals correspond to increased GDOP values, primarily due to the satellites not being fully aligned with the zenith. This misalignment significantly impacts the geometric configuration, making any abnormal data from a single satellite more influential on the positioning results, particularly when there is a limited amount of observational data. This further indicates that, under poor satellite geometric configurations, the accuracy of the model corrections for the current epoch cannot be guaranteed, subsequently reducing the confidence in the positioning results.

### 3.2. Observation Residuals

**Figure 3:**
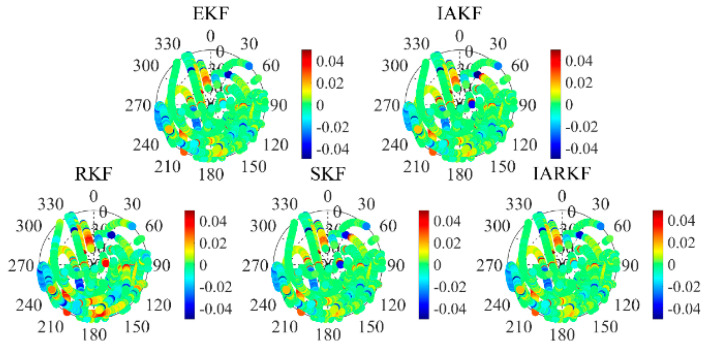
Comparison of posterior residuals in the sky vision.

In the azimuth dimension, we observed an uneven distribution of residuals in the five sky plots of the micro–nano satellite data from 2023. Specifically, a notable gap in residuals was identified in the azimuth range from 270° to 60°. The reception of GPS satellite signals in this azimuth range is significantly affected, indicating that the blank areas in the residuals are closely related to satellite rotation. The analysis of satellite data is further detailed in Figure 3.

Additionally, the elevation angle has a significant impact on the residuals; as the elevation angle increases, the distribution of posterior residuals tends to be more consistent and approaches zero. Satellites observed at higher elevation angles typically provide more reliable observational data, resulting in more accurate triangulation measurements. In contrast, signals from satellites at low elevation angles are influenced by atmospheric delay errors and severe multipath effects, making precise corrections challenging and leading to decreased data accuracy. This observation highlights the need for controlling signal quality in general missions by setting a reasonable cutoff elevation angle for satellites.

When the number of tracked satellites is low, the geometric layout of the navigation satellites becomes poor, resulting in larger positioning errors. Moreover, since low Earth orbit satellites operate at relatively low altitudes, they are closer to the Earth’s surface where atmospheric drag and other factors can lead to greater observational errors and more complex dynamics model inaccuracies, ultimately impacting orbit determination accuracy [21]. To enhance the accuracy of the analysis, this paper excludes outlier residual data, allowing for a clearer illustration of the characteristics of different filtering algorithms.

**Figure 4:**
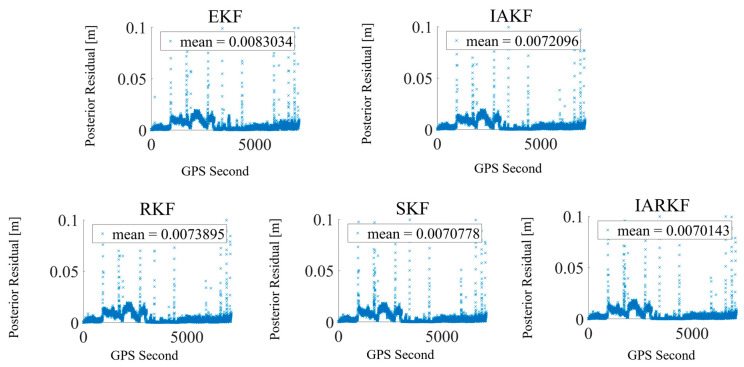
Comparison of posterior residual scatter.

As shown in Figure 4, the posterior residual for the EKF is 0.0083034 m, while the values for IAKF, RKF, SKF, and IARKF are 0.0072096 m, 0.0073895 m, 0.0070778 m, and 0.0070143 m, respectively. Compared to the EKF, SKF demonstrates a 14.8% improvement, highlighting its advantage in enhancing the numerical stability of the filter. By applying square root processing to the covariance matrix, the SKF improves numerical stability and consistency of the residuals. The IAKF shows a 13.2% improvement; its iterative adaptive mechanism dynamically adjusts noise covariance parameters, optimizing filtering accuracy, particularly in dynamic noise scenarios. While slightly lower than the IARKF and SKF, the IAKF still significantly enhances the consistency of posterior residuals.

The RKF has a smaller improvement of 10.9%. Although it optimizes residuals through weight adjustments and multiple iterations, its effectiveness is less pronounced than that of IARKF and SKF, potentially due to its weaker resistance to continuous errors. The IARKF achieves the greatest consistency improvement in posterior residuals, reaching 15.5%. This indicates that the IARKF exhibits the strongest performance in processing actual data, particularly in reducing residuals and enhancing accuracy. Its adaptive adjustment mechanism effectively addresses noise and data anomalies, resulting in the most significant reduction in posterior residuals.

Overall, the IARKF performs best in terms of reducing the consistency of posterior residuals, showcasing its robust adaptive capability and effectiveness in handling outlier data, making it optimal for practical applications. The SKF and IAKF also significantly enhance residual consistency, reflecting their advantages in stability and accuracy optimization. Although the RKF shows a smaller improvement, it still outperforms the EKF, indicating its capability in handling specific noise conditions.

The increase in residuals in Figure 4 is mainly due to the data processing approach, which prioritizes maximizing data utilization by retaining all available observations, including those from low-elevation satellites. These lead to joint gross errors, which our algorithm corrects through time. Additionally, when the number of observation satellites or data points changes rapidly, newly added satellites can cause temporary instability due to sensor calibration and state estimation; however, these fluctuations are short-lived and rare, with our algorithm quickly compensating through ongoing updates.

### 3.3. Overlapping Arc Error Analysis

This analysis focuses on the time period from 19 July 2023 00:04:08 to 00:30:31, totaling approximately 26 min. During this interval, the orbit determination algorithm meets the theoretical convergence conditions. Although the observational data within the overlapping arc segment are consistent, the differing start and end epochs of the orbit determination allow this overlapping section to be utilized for evaluating the consistency of the orbit determination algorithm during this time frame. This, in turn, provides an indirect verification of the algorithm’s stability and accuracy.

**Figure 5:**
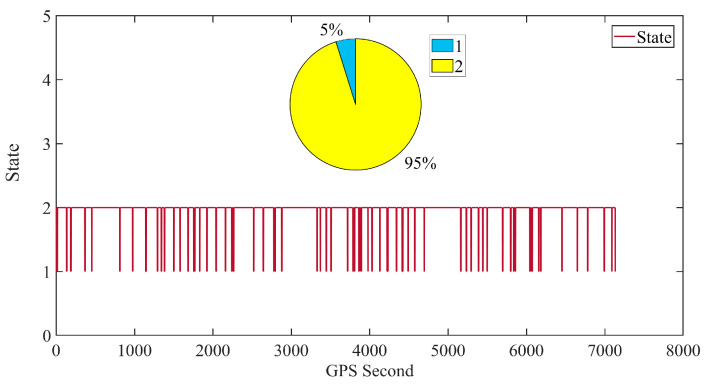
Filtering level variation curve and distribution map.

Observing State 2 in Figure 5, it can be noted that even with a limited number of satellites during this segment of orbit determination, the IARKF still achieves a high coverage rate, with only a small amount of time spent in a degraded state. This demonstrates that the strategy effectively maximizes its advantages even in suboptimal working conditions.

In the orbital data, the primary sources of error include ephemeris errors and the a priori noise matrix in the observations. Ephemeris errors arise from inaccuracies in the ephemeris model and prediction errors, directly affecting orbital calculation precision and potentially leading to orbital deviations and reduced tracking accuracy, which can impact mission planning and satellite navigation. Larger a priori noise in the observations disrupts the prediction and update phases of the filter, causing errors to propagate and accumulate, thus lowering the final accuracy of the orbital solution.

**Figure 6:**
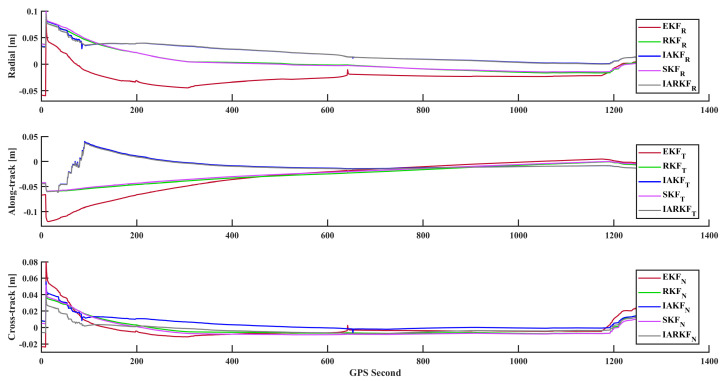
Comparison of errors for overlapping arc.

Analyzing Figure 6 and Table 3, the RKF, IAKF, SKF, and IARKF improve 3D errors by approximately 27.3%, 38.4%, 29.2%, and 40.2% compared to EKF, respectively. It is observed that all methods exhibit some degree of accuracy spikes, indicating positional anomalies at certain epochs, which result in inconsistent deviations between two orbit determination results. Notably, the IARKF responds the fastest during anomalous intervals, with a smoother convergence curve and no significant systematic errors. This is primarily due to IARKF’s use of exponentially decaying memory weighting, which replaces traditional average weighting and increases the importance of the latest information, enhancing tracking timeliness and reducing algorithm response times.

In contrast, the other four filtering methods show varying degrees of positional bias; however, there are no significant differences in normal positioning arcs among the five methods. This is mainly because the improved algorithms are optimized for handling exceptional situations, enhancing the robustness and outlier resistance of the algorithms themselves. Notably, all five methods achieve a 3D overlapping arc RMS error of less than 1 dm, with the IARKF reaching 3.14 cm, demonstrating its stability and effectiveness under relatively harsh observational conditions while providing algorithmic support for high-precision orbit determination during subsequent satellite stabilization phases.

## 4. Verification of the Stable Earth-Pointing Scenario for In-Orbit Satellite

To validate the orbit determination accuracy of the proposed algorithm during the earth-pointing stabilization phase, we selected a set of data from the satellite in 2024 under stable conditions. The total available arc length for orbit determination is 1.8 h. Specifically, we selected the first segment of orbital data from 8 July 2024 23:17:50 to 9 July 00:47:50 (with an arc length of 1.5 h), and the second segment from 9 July 23:47:50 to 10 July 01:05:57 (with an arc length of 1.3 h), resulting in an overlapping arc segment of 1 h between the two datasets.

### 4.1. GDOP

**Figure 7:**
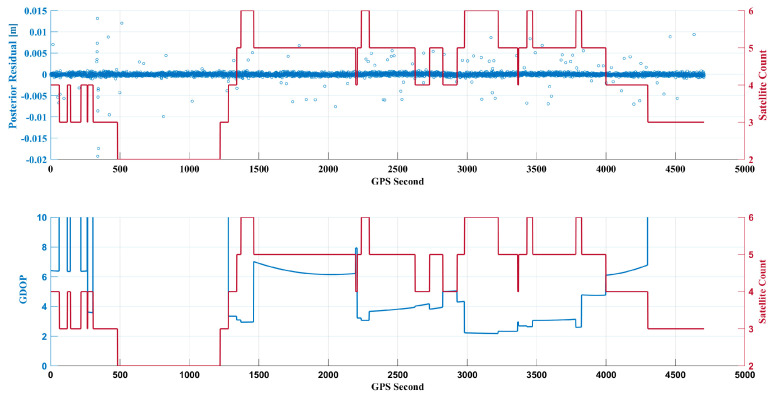
Comparison of posterior residuals, number of positioning satellites, and GDOP.

Compared with Figure 2, it is evident in Figure 7 that the frequency of variation in combined-frequency GDOP has significantly decreased, with an increased proportion of smooth data segments. This visual representation clearly indicates the absence of significant satellite rotation phenomena.

As shown in Figure 7, during the stable earth-pointing phase of the micro–nano satellite, the proportion of instances with five or more satellites reaches 71%, with an average of 4.9 satellites participating in positioning. The average GDOP value is 1.7393 and the average posterior residual for the channels is 0.0038 m. In comparison, the average GDOP value using the nulling combination in a rotating state is 2.5683, indicating a reduction to 1.7393 in the stabilized state, which represents a decrease of approximately 32.3%. This stable earth-pointing attitude aids in the reception and processing of signals, significantly reducing observation noise caused by attitude changes, thereby improving data quality and enhancing the accuracy and reliability of orbit determination.

**Figure 8:**
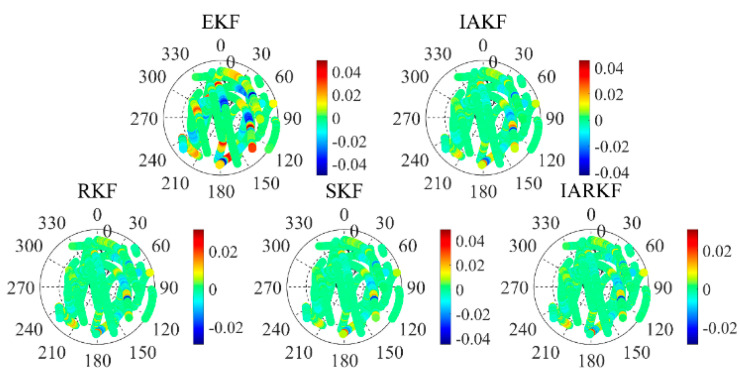
Comparison of posterior residuals in the sky vision.

Although the average number of positioning satellites is lower than the slow rotation scenario, which had an average of 5.3 satellites, the GDOP remains low and stable. Observing Figure 7, it can be seen that most posterior residuals are concentrated below 10 cm, with noticeably fewer anomalous residuals. In intervals where the number of satellites is below four, the corresponding GDOP values increase significantly, leading to greater uncertainty in the positioning results. In the absence of observational data, the filter relies on previous orbital states and model predictions to estimate the current state. Without new observational data to correct and update the state estimates, the predictive errors primarily stem from the orbital model, which accumulates and becomes more significant, leading to a noticeable dispersion trend in the residual scatter, presented in Figure 8.

The trend of the number of satellites changes gradually, indicating a significant decrease in rotation frequency and a normal geometric distribution of satellites within the field of view.

### 4.2. Observational Residuals

Compared to the slow rotation scenario, the proportion of large residuals in the stable earth-pointing scenario is significantly lower. This is primarily due to the positive correlation between signal observation quality and elevation angle. Low-elevation-angle satellite observations not only suffer from larger atmospheric delay correction errors but are also more susceptible to severe multipath effects. Typically, a cutoff elevation angle is set to avoid such satellites. For GPS satellites, a relatively low cutoff elevation angle (around 15°) is often chosen to increase the number of available satellites while simultaneously enhancing the quality of the signals used for orbit determination.

**Figure 9:**
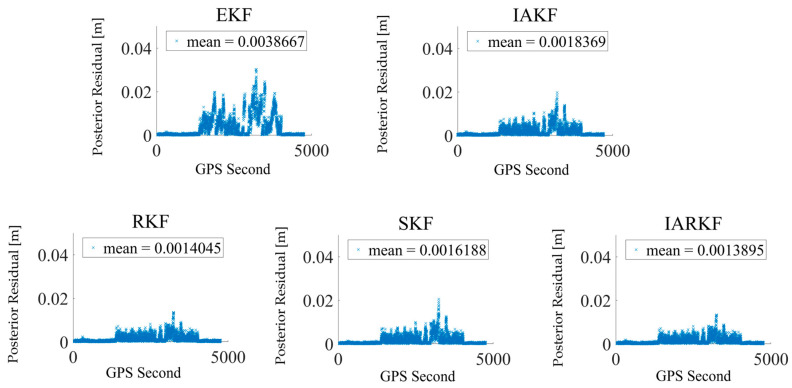
Comparison of posterior residual scatter.

As shown in Figure 9, the posterior residual for the EKF is 0.0038667 m, while the residuals for IAKF, RKF, SKF, and IARKF are 0.0018369 m, 0.0014045 m, 0.0016188 m, and 0.0013895 m, respectively. This represents reductions of 52.5%, 63.7%, 58.2%, and 64.0%, with the IARKF demonstrating the greatest improvement.

Overall, the IARKF exhibits the best performance in reducing the consistency of posterior residuals, thanks to its robust adaptive capabilities and effectiveness in handling anomalous data. However, in this dataset, the RKF shows significant improvements compared to the IAKF and SKF. This can be attributed to RKF’s inherent algorithmic structure, which adjusts weights based on the distribution of residuals and iteratively minimizes these residuals. The distribution of outliers in the figure is notably reduced, and the RKF effectively suppresses discrete anomalies.

Both the SKF and IAKF also demonstrate advantages in stability and precision optimization. Their weight update mechanisms are relatively fixed, providing stable performance. However, due to the reduced continuous noise in non-rotating satellite data, the improvements in orbit determination accuracy using these two algorithms in the stable earth-pointing scenario are modest. The IARKF, on the other hand, shows the largest improvement, and the significant reduction in the number of anomalous points in the residuals corresponds well with the RKF results, underscoring IARKF’s effectiveness in addressing various anomalies in real on-orbit data.

### 4.3. Overlapping Arc Segment Error Analysis

The analysis of overlapping arc segments begins on 9 July at 00:28:42, when the theoretical convergence of the orbit determination is achieved, and concludes at 00:59:03, covering a total duration of approximately 30 min.

**Figure 10:**
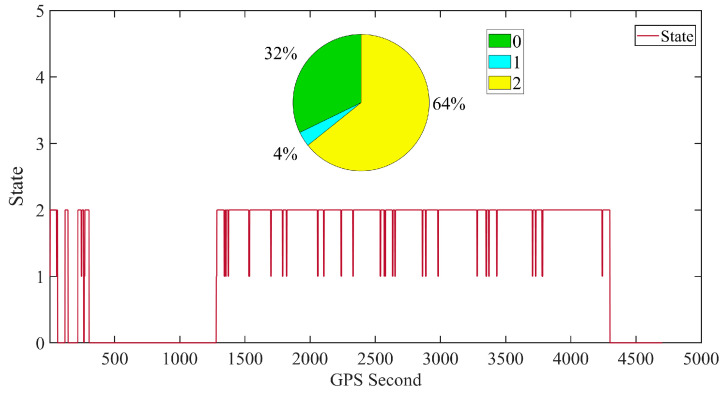
Filtering level variation curve and distribution map.

Observing the proportions of various states in Figure 10, it is evident that during this segment with a stable number of tracking satellites, the IARKF achieves a high coverage rate, with only minimal time downgraded to the ARKF. In instances where the number of satellites is anomalous, the algorithm automatically and accurately identifies the need to downgrade to the EKF state (0 state) due to the lack of high-quality observational data for correction. This prioritization of maintaining continuous orbit determination demonstrates that the strategy can effectively leverage its advantages even in less-than-ideal operational environments.

**Figure 11:**
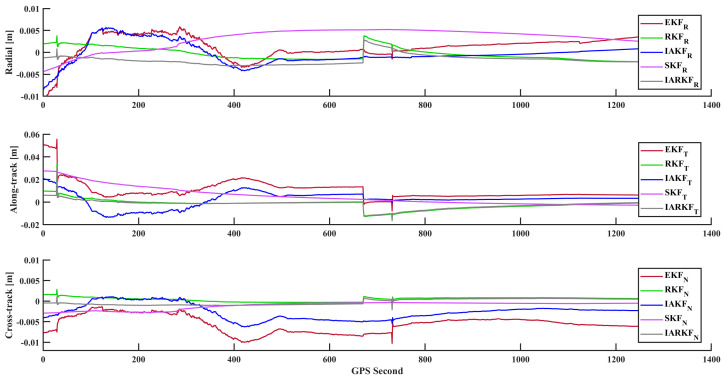
Comparison of errors for overlapping arc.

By analyzing Figure 11 and Table 4, using the same observational data, the RKF, IAKF, SKF, and IARKF achieve improvements in 3D error of approximately 57.8%, 32.8%, 12.9%, and 60.3% compared to EKF, respectively. The IAKF and SKF exhibit similar positioning accuracy, while the RKF performs better than both and approaches the accuracy of the IARKF. This indicates that in this dataset, the majority of anomalous errors are predominantly discrete measurement values, and the continuous gradual errors primarily related to ionospheric effects have been largely mitigated through the ionospheric cancellation combination.

Observing the IAKF, its tracking curve shows significant fluctuations, making it challenging to accurately estimate the magnitude of isolated disturbances. This leads to a mismatch between the estimated and actual measurement noise variance, consequently reducing positioning accuracy. In environments where measurement noise and dynamical model errors vary significantly, convergence may be slower, and excessive adjustments during iterations can introduce unnecessary fluctuations.

In contrast, IARKF excels in both positioning stability and accuracy. It dynamically adjusts the noise covariance matrix and optimizes filtering parameters in real time based on observational data. Its adaptive adjustments to the innovation sequence effectively reduce the negative impact of outliers on filtering results, enabling the algorithm to respond robustly to various environmental changes and data anomalies, thereby enhancing filtering accuracy and convergence speed.

## 5. Conclusions

This study addresses the inherent limitations of GNSS receivers on micro- and nano-satellites, particularly in complex dynamic scenarios involving variations in satellite attitude during specific missions. We propose an innovative Hierarchical Robust Adaptive Kalman Filtering (IARKF) algorithm to enhance robustness and precision in such challenging environments.

Initially, we introduce an adaptive factor to tackle typical observational errors, drawing on the principles of adaptive robust filtering to dynamically adjust the distribution of the innovation vector. This adaptive adjustment effectively mitigates errors arising from environmental changes or system dynamics, thereby improving the robustness and stability of the filter.

Furthermore, we employ a diminishing memory weighting method for real-time noise estimation. This approach processes historical observational data with weighted significance, gradually reducing the impact of outdated data on current estimates, thus enhancing the accuracy and reliability of noise estimation. The diminishing memory weighting method enables real-time reflection of system state changes, bolstering the filter’s adaptability to dynamic conditions.

Additionally, we propose a segmented adaptive filtering strategy to address various filtering scenarios. This strategy divides the overall filtering process into multiple phases, each utilizing distinct filtering parameters and strategies, thereby optimizing performance under different operational conditions. Such a segmented approach not only enhances the flexibility of the filter but also strengthens its efficacy in complex environments.

Through comparative analysis of on-orbit data from the micro–nano satellite in both slow-rotation and stable earth-pointing scenarios, we demonstrate that the IARKF algorithm excels in precision enhancement. Compared to traditional Extended Kalman Filtering, the IARKF improves positioning accuracy by 40.2% and 60.3% in slow-rotation and stable earth-pointing scenarios, respectively. Moreover, in stable conditions, the IARKF achieves an approximate 85.3% improvement over its performance in slow-rotation scenarios. These findings indicate that the IARKF algorithm not only enhances trajectory precision but also bolsters robustness in complex dynamic environments, providing critical insights for future high-performance trajectory applications in micro- and nano-satellites.

## Figures and Tables

**Table 1:** Orbital modeling and geodetic framework setup.

Item	Relevant Setting
Dynamic Model	
Earth Gravity Field	JGM3 (20 × 20) (Tapley et al., 1996)
Gravitational Effects of Celestial Bodies	Simplified Analytical Model of Moon and Sun (Montenbruck and Pfleger, 2013)
Solid Earth Tides	Neglected
Ocean Tides	Neglected
Polar Tides	Neglected
Solar Radiation Pressure	Cylindrical Shadow Model (Montenbruck et al., 2002)
Atmospheric Drag	Harris–Priester Model (Density) (Cappellari et al., 1976)
Earth Radiation Pressure and Relativity Effects	Neglected
Reference Frame	
Coordinate System	J2000/WGS84 Coordinate System
Precession and Nutation	IAU 1976/IAU-1980 Simplified Model

**Table 2:** GNSS data processing strategy.

Item	Relevant Setting
GNSS Signal	GPS L1C + L2C
Combination	Undifferenced Dual-frequency Ionosphere-free Combination
GNSS Orbit and Clock	Broadcast Ephemeris
Ambiguity	Float Resolution
Elevation Cutoff	Neglected
Sampling Rate	1 Hz

**Table 3:** Accuracy comparison of overlapping arc.

**Filtering Algorithm**	**Position Error in X Axis/m**	**Position Error in Y Axis/m**	**Position Error in Z Axis/m**	**3D Error/m**
EKF	0.0119	0.0269	0.0435	0.0525
RKF	0.0094	0.0140	0.0342	0.0381
IAKF	0.0123	0.0242	0.0176	0.0324
SKF	0.0100	0.0149	0.0325	0.0371
IARKF	0.0174	0.0199	0.0170	0.0314
**Filtering Algorithm**	**Position Error in X Axis/m**	**Position Error in Y Axis/m**	**Position Error in Z Axis/m**	**3D Error/m**
EKF	0.0271	0.0432	0.0125	0.0525
RKF	0.0211	0.0301	0.0010	0.0381
IAKF	0.0262	0.0165	0.0095	0.0324
SKF	0.0218	0.0282	0.0105	0.0371
IARKF	0.0258	0.0166	0.0068	0.0314

**Table 4:** Accuracy comparison of overlapping arc.

**Filtering Algorithm**	**Position Error in X Axis/m**	**Position Error in Y Axis/m**	**Position Error in Z Axis/m**	**3D Error/m**
EKF	0.0079	0.0058	0.0061	0.0116
RKF	0.0020	0.0033	0.0030	0.0049
IAKF	0.0051	0.0045	0.0038	0.0078
SKF	0.0065	0.0073	0.0028	0.0101
IARKF	0.0019	0.0031	0.0028	0.0046
**Filtering Algorithm**	**Position Error in X axis/m**	**Position Error in Y axis/m**	**Position Error in Z axis/m**	**3D Error/m**
EKF	0.0029	0.0127	0.0059	0.0116
RKF	0.0015	0.0046	0.0007	0.0049
IAKF	0.0024	0.0067	0.0032	0.0078
SKF	0.0038	0.0093	0.0014	0.0101
IARKF	0.0020	0.0040	0.0007	0.0046

## Data Availability

Data are unavailable due to privacy restrictions.

## Appendix A

**Table A1:** Satellite status information for civilian signal broadcast.

PRN	Satellite	Launch Date (UTC)	Positioning Signals
13	GPS 2R-2	1997/7/23	L1C/A
20	GPS 2R-4	2000/5/11	L1C/A
22	GPS 2R-5	2000/7/16	L1C/A
16	GPS 2R-8	2003/1/29	L1C/A
21	GPS 2R-9	2003/3/31	L1C/A
19	GPS 2R-11	2004/3/20	L1C/A
2	GPS 2R-13	2004/11/6	L1C/A
17	GPS 2R-14M	2005/9/26	L1C/A, L2C
31	GPS 2R-15M	2006/9/25	L1C/A, L2C
12	GPS 2R-16M	2006/11/17	L1C/A, L2C
15	GPS 2R-17M	2007/10/17	L1C/A, L2C
29	GPS 2R-18M	2007/12/20	L1C/A, L2C
7	GPS 2R-19M	2008/3/15	L1C/A, L2C
5	GPS 2R-21M	2009/8/17	L1C/A, L2C
25	GPS 2F-1	2010/5/28	L1C/A, L2C, L5
24	GPS 2F-3	2012/10/4	L1C/A, L2C, L5
27	GPS 2F-4	2013/5/15	L1C/A, L2C, L5
6	GPS 2F-6	2014/5/17	L1C/A, L2C, L5
9	GPS 2F-7	2014/8/2	L1C/A, L2C, L5
3	GPS 2F-8	2014/10/29	L1C/A, L2C, L5
26	GPS 2F-9	2015/3/25	L1C/A, L2C, L5
8	GPS 2F-10	2015/7/15	L1C/A, L2C, L5
10	GPS 2F-11	2015/10/31	L1C/A, L2C, L5
32	GPS 2F-12	2016/2/5	L1C/A, L2C, L5
4	GPS 3-1	2018/12/23	L1C/A, L2C, L5
18	GPS 3-2	2019/8/22	L1C/A, L2C, L5
23	GPS 3-3	2020/6/30	L1C/A, L2C, L5
14	GPS 3-4	2020/11/5	L1C/A, L2C, L5
11	GPS 3-5	2021/6/17	L1C/A, L2C, L5
28	GPS 3-6	2023/1/18	L1C/A, L2C, L5

The status information of the satellites broadcasting civilian signals is sourced from the Navigation Center, U.S. Department of Homeland Security.
